# Analysis of SLC7A9 gene mutations among Jordanian patients with cystinuria

**DOI:** 10.1016/j.amsu.2021.102182

**Published:** 2021-02-25

**Authors:** Omar M. Halalsheh, Mustafa A. Al-Shehabat, Moh''D.A. Al-Ghazo, Ibrahim F. Al-Ghalayini, Yaman A. Altal, Radwan Al-Okour, Omar Altal

**Affiliations:** a Department of General Surgery and Urology, Faculty of Medicine, Jordan University of Science and Technology, Irbid, 22110, Jordan; b Department of Physiology and Biochemistry, Faculty of Medicine, Jordan University of Science and Technology, Irbid, 22110, Jordan; c Department of Obstetrics and Gynecology, Faculty of Medicine, Jordan University of Science and Technology, Irbid, 22110, Jordan

**Keywords:** Cystinuria, Mutations, SLC7A9, Gene

## Abstract

**Background:**

Cystinuria is known as a heritable disorder affecting the cysteine reabsorption by renal system as well as the reabsorption of dibasic amino acids. The main objectives of the present study were to identify genetic mutations in SLC7A9 gene associated with cystinuria.

**Methods:**

A cross sectional study design was conducted. A total of 28 patients diagnosed with cystinuria were included. Molecular techniques were applied to identify genetic mutations in SLC7A9 gene.

**Results:**

The mean age of study participants was 31.57 ± 2.88 years, and slightly more than two thirds of participants were males. Mutations of SLC 7A9 gene showed that the majority of cases (57.1%) were homogeneous, (7.1%) heterogeneous, and slightly more than one third of patients had no mutations. There was no statistically significant relationship between mutations for the SLC7A9 gene and gender (p = 0.249).

**Conclusion:**

Mutations in the SLC7A9 gene are prevalent and can be used as molecular tools to diagnose cystinuria.

## Highlights

• Cystinuria is an inherited disorder of dibasic amino acid as cystine transport in kidney.
• SLC7A9 Cystinuria gene was recently isolated in many cystinuria patient from different racial populations.
• Mutations in the SLC7A9 gene are prevalent in Jordanian (Arab) populations.

## 1 Introduction

Cystinuria is known as a heritable disorder affecting the cysteine reabsorption by renal system as well as the reabsorption of dibasic amino acids [1]. The underlying cause for cystinuria is the alteration in transporting mechanisms of cystine, arginine, lysine, and ornithine in renal system and the intestinal tract [2]. It has been shown that the transport is facilitated by the rBAT/b0,þ AT transporter. The subunits for this transporter are programmed by the genes SLC3A1 and SLC7A9 [[3], [4], [5]].

The rBAT-b0,+ AT transporter, composed of the SLC3A1 and SLC7A9 protein subunits, is identified as dibasic amino acid reabsorption transporter. The SLC3A1 subunit encodes for rBAT protein while the SLC7A9 subunit encodes for the bo,+, AT [3[. About 90% of cystine reabsorption is accounted for this transporter [6]. In a healthy individual, dibasic amino acids including cystine are filtered by the glomerulus and are reabsorbed across the apical membrane of the proximal tubule through the rBAT-b0,+ AT transporter. Once cystine and the dibasic amino acids are transported in the proximal tubule cell, intracellular cystine is readily reduced to two molecules of cysteine (Fig. 2). In a cystinuric patient, cystine is not reabsorbed through the rBAT-b0,+ AT transporter and therefore, accumulates in the urine, leading to stone formation.

Cystinuria is classified into type I, II and III. This classification is based on the biochemical determination of urinary cystine hyperexcretion in the patients’ parents [7]. Utilizing the molecular tools showed that genetic mutations of SLC3A1 were associated with type I cystinuria, while mutations of SLC7A9 were associated with non-type I cystinuria [8].

The main objectives of the present study were to identify genetic mutations in SLC7A9 gene associated with cystinuria in Jordanian individuals.

## 2 Methods and subjects

After obtaining the ethical approval from the ethical committee of Jordan University of Science and Technology. A cross sectional study design was conducted at Urology Department of King Abdulla University Hospital which is a tertiary hospital affiliated to Jordan University of Science and Technology. The study target was to include all patients diagnosed with cystine stones at our center from 2017 to 2020. Twenty-eight patients were recognized, a written informed consents were collected, and basic demographic data was obtained.

After that, a total of 3 ml of whole blood samples (assigned samples for chemistry and hematology tubes) were withdrawn from each participant, centrifuged for 20 min, and the samples were stored at −20C until used.

DNA was extracted from the whole blood using Promega Genomic DNA purification kit (Promega,USA). Three ml of blood was added to 9 ml of cell lysis solution to lyse the red blood cells (RBCs). This is followed by the addition of 3 ml of nuclei lysis solution to lyse the white blood cells (WBCs) cells. Digests were deproteinized by addition of 1 ml of protein precipitation solution. The DNA then was salting out by the addition of isopropanol. The precipitated DNA was then washed using 70% ethanol and dehydrated using DNA rehydration solution.

In addition, urine samples were collected from patients to measure cystine levels and compare it to the average values which is presented in the supplementary table.

Gel electrophoresis was used in order to visualize the extracted DNA. 5 μl of extracted genomic DNA was mixed with 2 μl of (10X) loading dye and loaded on a 2% agarose gel (1X). Tris Base EDTA (TBE) buffer was used to perform the gel electrophoresis at constant voltage (100V) for 1.5 h. Then, the gel was stained with ethidium bromide (1 mg/L) and visualized by gel document (BioRad, USA) to photograph the gel.

Data was entered into SPSS statistical analysis software (SPSS, version 19.0, SPSS Inc., Chicago, IL, USA). Data was represented as frequencies and percentages for categorized variables, means and standard deviations for numerical variables (mean ± SD).

This study was conducted according to the Strengthening the reporting of cohort studies in surgery (STROCSS) 2019 Guideline [9].

## 3 Results

Table 1 showed that the mean age of study participants was 31.6 years, and slightly more than two thirds of participants were males. Mutations of SLC7A9 gene showed that the majority of the cases (57.1%) were homogeneous, (7.1%) were heterogeneous, and slightly more than one third of patients had no mutations.

**Table 1:** General characteristics of participants.

Pre- and post-operative Variables	Number	Percent (%)
	Mean ± SD	
**Sex**		
Male	19	67.9
Female	9	32.1
**Age (y)**	31.57 ± 2.88	
**Side of stones**		
Bilateral	19	67.9
Right	6	21.4
Left	3	10.7
**Type of mutation in SLC 7A9 gene**		
Normal	10	35.7
Heterogenous	2	7.1
Homogenous	16	57.2

There was no statistically significant relationship between mutations of the SLC7A9 gene and the gender or age.

As shown in Table 2, the readings of dibasic amino acids in urine for 20 cystinuria patients were significantly high for cystine when compared to the normal average.

**Table 2:** The measured dibasic amino acids for 20 patients.

Patient name	Cystine	Ornthine	Lysine	Argnine
1	2803	8935	3168	6322
2	1809	1854	5861	6993
3	1357	1616	5991	4990
4	2127	1347	6115	3601
5	47	0	149	0
6	1628	1501	4542	2571
7	1446	1621	4443	2876
8	1711	2124	5270	6321
9	1994	2557	6779	5446
10	1305	1111	3707	1818
11	725	1178	3005	3740
12	1519	1515	5047	3821
13	353	0	2499	347
14	794	0	4945	2038
15	12280	7900	37580	22000
16	2069	2537	6120	4483
17	2390	1822	7829	4269
18	4155	3791	13806	0
19	1525	3510	10244	6521
20	1072	2335	4599	3634

**Fig. 1:**
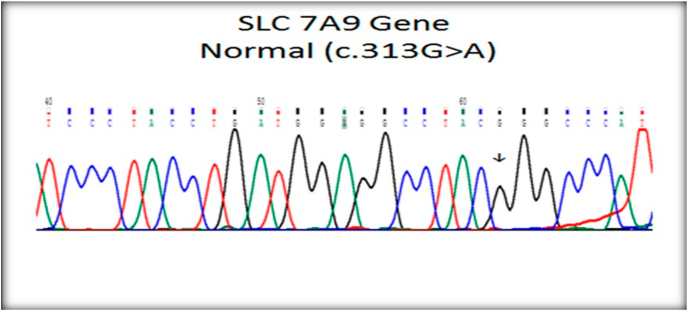
Normal SLC 7A9 gene.

**Fig. 2:**
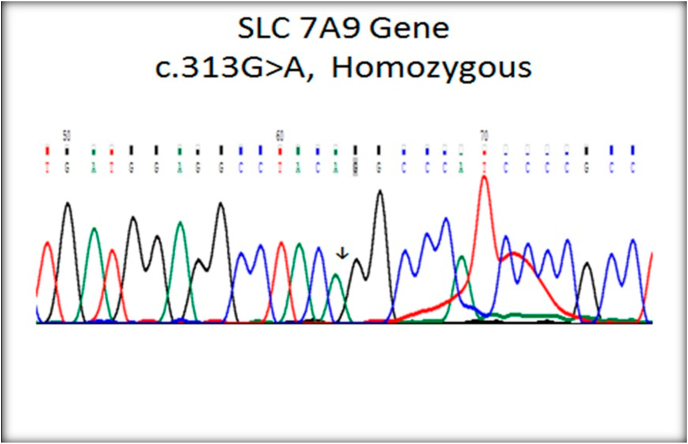
Homozygous SLC 7A9 gene.

**Fig. 3:**
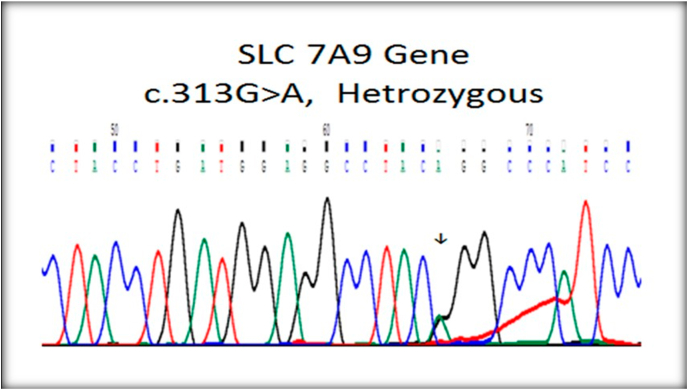
Heterozygous SLC 7A9 gene.

Fig. 1, Fig. 2, Fig. 3 showed the sequencing results including DNA sequencing and verifying the presence of genetic mutation in the patients proved by presence of cystine in urine.

## 4 Discussion

Screening of cystinuria among families was carried out in northern Jordan and a high prevalence was reported [10]. This study was limited and lacked the genetic evaluation of disease. Unfortunately, up-to- date literature has no evidence for any genetic study of this disease among Arabs at all.

Ata and Jaradat conducted a study to identify the genetic bases of this disease among Jordanian patients [11]. They studied 24 unclassified cystinuria patients from 14 unrelated families. They started by screening for three mutations (M467T, T216 M and E483X), described more than once in Mediterranean populations by RFLP technique [11]. None of these common mutations was detected in patients. The result is in concordance with the suggestion of many studies, that ethnic origin might indicate the population specific mutations responsible of disease [12].

Due to diversity of mutations among different populations, a strategy of pre-screening of all exons, exons/intron boundaries by SSCP (single nucleotide conformational polymorphism), followed by sequencing was sufficient to detect variants and recommended for establishing a diagnostic test [13]. SSCP is a commonly used mutation scanning technique, but its sensitivity is being with fragments less than 300 bp. direct sequencing of DNA is the most sensitive technique compared to other molecular techniques for detecting of variants, even relatively expensive. Using DNA direct sequencing, many novel mutations and SNPs were reported [5]. In the same study, direct sequencing of all coding regions and splice junctions of SLC3A1 gene was carried out. Five mutations and 4 polymorphisms, Y461H were detected and these were previously reported missense mutations in Americans [14]. It causes T to C nucleotide substitution at nucleotide position 1381, changed (TAT) codon of amino acid Tyrosine (uncharged polar side chain) to (CAT) codon of amino acid Histidine (basic side chain) at position 416. This amino acid Tyrosine was shown to be conserved at this position in human, rat and rabbit, implying its importance in protein activity. Another missense mutation R456C was found, it causes substitution of nucleotide C at position 1366 by T. The normal codon (CGT) corresponding to Arginine (basic side chain) was changed to (TGT), corresponding to Cysteine (uncharged polar side chain) [15].

Cystinuria is an autosomal recessive disorder that occurs as the result of mutations in one of two genes that code for the proteins that constitute the dibasic amino acid including cystine transporters expressed in the proximal renal tubules [16], which results in the failure to reabsorb filtered cystine, a poorly soluble amino acid that crystallizes in the distal tubules and forms large and recurrent stones.

The results of the present study showed that the detected mutations of SLC 7A9 gene were homogeneous in the majority of cases (75.1%) and heterogeneous mutations were (7.1%). More than one third of patients had no mutations. However, previous studies indicated to the existence of 30 mutations in SLC7A9 among cystinuria patients [[17], [18], [19]].

The results of this study did not show a significant relationship between mutations in SLC7A9 and gender (p > 0.05), although males tended to have more homogeneous mutations compared with females. This finding is consistent with other studies in which males are more affected than males by cystinuria [7,20].

This study is not without limitation. The small sample size is one of the limitations. Also, the small number of confounding factors is another limitation. Moreover, many other mutations were studied and not applied to our study.

## 5 Conclusion

Mutations in the SLC7A9 gene are prevalent and can be used as molecular tools to diagnose cystinuria. Most of the mutations were homogenous and there is no difference between males and females.

## Funding

The authors have not declared any grant for this work from any funding authority.

## Declaration of competing InterestCOI

The authors have no financial ties or conflicts of interest to disclose.

## Ethical approval

This study has been performed in accordance with the ethical standards laid down in the 1964 Declaration of Helsinki and its later amendment. This research has obtained ethical approval from Research and Ethics Committee, at Jordan University of Science and Technology and King Abdullah University Hospital, Irbid, Jordan.

## Consent

Written informed consent was obtained from each patient.

## Author contribution

All authors contributed significantly and in agreement with the content of the article. All authors were involved in project design, data collection, analysis, statistical analysis, data interpretation and writing the manuscript. All authors presented substantial contributions to the article and participated of correction and final approval of the version to be submitted.

## Registration of research studies

Researchregistry6400.

## Guarantor

Omar Halalsheh.

## Data availability

The dataset generated and analyzed during the current study is available from the corresponding author on reasonable request.

## Provenance and peer review

Not commissioned, externally peer-reviewed.
