# Supplementary material for: Analysis of SLC7A9 gene mutations among Jordanian patients with cystinuria
Source: Ann Med Surg (Lond). 2021 Feb 25;63:102182. doi: 10.1016/j.amsu.2021.102182 (PMC7930589; doi:10.1016/j.amsu.2021.102182)
Supplement: Multimedia component 1 [file mmc1.doc]

**Amino acids in urine**

| **Amino Acids** | **Normal Ranges**  **premature**  **(umol/L)** | **Normal Ranges**  **(0-1) months**  **(umol/L)** | **Normal Ranges (1-24) months**  **(umol/L** | **Normal Ranges (2-18) years**  **(umol/L** | **Normal**  **Ranges more than 18 years**  **(umol/L** |
| --- | --- | --- | --- | --- | --- |
| **Taurine** | **5190-23620** | **1650-6220** | **545-3790** | **639-1866** | **380-1850** |
| **Citrulline** | **240-1320** | **27-181** | **22-180** | **10-99** | **8-50** |
| **Aspartic acid** | **580-1520** | **336-810** | **230-685** | **0-120** | **60-240** |
| **Threonine** | **840-5700** | **445-1122** | **252-1528** | **121-389** | **130-370** |
| **Serine** | **1680-6000** | **1444-3661** | **845-3190** | **362-1110** | **240-670** |
| **Aspargine** | **1350-5250** | **185-1550** | **252-1280** | **72-332** | **99-470** |
| **Glutamic acid** | **380-3760** | **70-1058** | **54-590** | **0-176** | **39-330** |
| **Glutamine** | **520-1700** | **393-1042** | **670-1562** | **369-1014** | **190-510** |
| **Aaa** | **70-460** | **0-180** | **45-268** | **2-88** | **40-110** |
| **Glycine** | **7840-23600** | **5749-16423** | **3023-11148** | **897-4500** | **730-4160** |
| **Alanine** | **1320-4040** | **982-3055** | **767-6090** | **231-915** | **240-670** |
| **aAminobutricacid** | **50-710** | **8-65** | **30-136** | **0-77** | **0-90** |
| **Valine** | **180-890** | **113-369** | **99-316** | **58-143** | **27-260** |
| **Cystine** | **480-1690** | **212-668** | **68-710** | **25-125** | **43-210** |
| **Methionine** | **500-1230** | **342-880** | **174-1090** | **16-114** | **38-210** |
| **Isoleucine** | **250-640** | **125-390** | **38-342** | **10-126** | **16-180** |
| **Leucine** | **190-790** | **78-195** | **70-570** | **30-500** | **30-150** |
| **Tyrosine** | **1090-6780** | **220-1650** | **333-1550** | **122-517** | **90-290** |
| **Phenylalanine** | **920-2280** | **91-457** | **175-1340** | **61-314** | **51-250** |
| **Homocystine** | **580-2230** | **0-88** | **6-67** | **0-32** | **0-32** |
| **Ornithine** | **260-3350** | **118-554** | **55-364** | **31-91** | **20-80** |
| **Lysine** | **1860-15460** | **270-1850** | **189-850** | **153-634** | **145-634** |
| **Histidine** | **1240-7240** | **908-2528** | **815-7090** | **644-2430** | **460-1430** |
| **1-methylhistidine** | **170-880** | **96-499** | **106-1275** | **170-1688** | **170-1680** |
| **Tryptophan** | **0** | **0** | **0-93** | **0-180** | **0-70** |
| **Arginine** | **190-820** | **35-214** | **38-165** | **31-109** | **10-90** |
| **proline** | **1350-10460** | **370-2323** | **254-2195** | **0** | **0** |
| **Hydroxyproline** | **560-5640** | **40-440** | **0-4010** | **0-3300** | **0-26** |
